# "You have to keep your nerve on a DMC." Challenges for Data Monitoring Committees in neonatal intensive care trials: Qualitative accounts from the BRACELET Study

**DOI:** 10.1371/journal.pone.0201037

**Published:** 2018-07-26

**Authors:** Claire Snowdon, Peter Brocklehurst, Robert C. Tasker, Martin Ward Platt, Diana Elbourne

**Affiliations:** 1 Department of Medical Statistics, London School of Hygiene and Tropical Medicine, University of London, London, United Kingdom; 2 Birmingham Clinical Trials Unit, Institute of Applied Health Research, University of Birmingham, Edgbaston, Birmingham, United Kingdom; 3 National Perinatal Epidemiology Unit, Nuffield Department of Population Health, University of Oxford, Oxford, United Kingdom; 4 Departments of Neurology and Anesthesia (Pediatrics), Harvard Medical School, Boston Children’s Hospital, Boston, Massachusetts, United States of America; 5 Division of Critical Care Medicine, Department of Anesthesiology, Perioperative and Pain Medicine, Boston Children’s Hospital and Harvard Medical School, Boston, Massachusetts, United States of America; 6 Newcastle Neonatal Service, Royal Victoria Infirmary, Newcastle upon Tyne, United Kingdom; Center of Pediatrics, GERMANY

## Abstract

**Background:**

Data Monitoring Committees (DMCs) are essential to the good conduct of many trials. Typically they comprise a small expert group which monitors safety, efficacy, progress and early outcome data as trials recruit. DMCs can recommend protocol revisions and early stopping of a trial. As DMC meetings usually consider unblinded interim data confidentially, their deliberations are seldom exposed to research scrutiny. Although there have been some case studies from trials from mixed specialties which offer insights into some of the common issues faced by DMCs, we have, however, little empirical information about the challenges faced within specific clinical settings.

**Methods:**

In-depth interviews with participants in the BRACELET Study on death and bereavement in neonatal intensive care trials produced qualitative accounts of experiences and views of a subgroup of 18 DMC members. These interviews explored views of DMC members in relation to the clinical context of neonatal intensive care and the conduct of neonatal intensive care trials.

**Results:**

Interviewees felt that an understanding of both the neonatal intensive care setting and population was crucial in a DMC. They considered the neonatal intensive care research population especially vulnerable, and that outcomes that included both death and severe disability raised particular challenges rarely faced in other settings. In exploring these key outcomes they were mindful of the need to meet high scientific standards and the needs of babies in the trials and their families. DMC members discussed particular difficulties around the composite outcome of death and severe disability, especially when mortality data were available long before data on longer term disability. While statistical stopping guidance is helpful, DMC members described decisions about stopping, revising or continuing a trial being informed by a wider set of considerations and discussions than a pre-set p value. These included potentially competing needs of current trial participants and future patients, and reflections on the nature of benefit and harm. Given their cognisance of the potential impact and consequences of the decisions made by DMCs in this setting of life, death, and disability, interviewees commonly used the imagery of bravery, and described DMCs either holding or losing their nerve.

**Conclusions:**

DMCs for trials in other fields may also face difficult ethical trade-offs in monitoring composite outcomes. The experience from this sample of DMC members suggest that for neonatal intensive care trials there are some very specific challenges seldom faced elsewhere. The vulnerability of the population, and the different timescales for essential data becoming available to inform decisions, presented particular challenges. We suggest that it is important to consider the challenges raised in other settings to better understand the complex work of these committees and to prepare future generations of DMC members.

## Background

Data Monitoring Committees (DMCs) are essential to the good conduct of many trials [1]. Typically they comprise a small group of individuals chosen for their clinical, experiential and methodological expertise. They monitor participant safety, evidence for efficacy or effectiveness, and can make recommendations to a Trial Steering Committee (TSC) about key actions for a trial. The DMC may recommend that a trial continues as per protocol, that there should be protocol revisions, or early stopping for futility, efficacy or harm. Each of these actions has important ramifications. In order to make their recommendations, DMC members are given access to interim and, where appropriate, unblinded data by a statistician who services the committee, information not usually available to any other individuals involved in the trial. The privileged information and the obligation they have to act on that information, place DMC members in a position of considerable trust, charging them with what has been described as a “substantial”[2] and “heavy” responsibility [3].

While we have a sizeable body of opinion-based literature to guide DMC methods and practice, we have few empirical data describing DMC member experiences, and in particular to explicate the nature and form of that substantial and heavy responsibility. DeMets and colleagues [4] gathered together first hand accounts of working practice in trials written by DMC members but there is little research-based literature. This may be because DMCs are less amenable to study than other research committees such as Research Ethics Committees (RECs) and TSCs which have received research attention[5][6][7]. As their meetings are usually closed, and members must maintain the confidentiality of proceedings, it is difficult to open DMCs to research scrutiny other than in generic and collective terms. The workings and nuanced challenges of individual trials can therefore be hard to uncover empirically and there is little to situate this experience within a specific specialty context. The remit, workings and constitution of DMCs have been the focus of collaborative efforts to produce guidelines for practice [8], but data-driven insights remain rare. One useful example is reported as part of the DAMOCLES Study in which a group of trials were surveyed and DMC members from a range of different clinical settings were interviewed [9]. The study also included a systematic review of DMC processes [10],and is the basis for the DMC Charter template [11] which is widely used, especially in public sector trials. One finding from DAMOCLES is particularly interesting. The study highlighted “courage” as a key component of DMC practice and suggested that “strong resolve” [3] is needed to manage difficult trial-related issues.

These images of heavy responsibility, courage and resolve suggest that DMC members develop and draw upon complex skill sets which go beyond implementing guidelines: Zuckerman and colleagues[2] suggest DMC members need “unique knowledge” of both DMC practice and clinical subject area to fulfil their role. DMC membership has been described as possibly “the toughest job in clinical medicine” (Drazen, cited by Harper [12]), but without empirical data exploring the work of DMCs, it can be difficult to understand the nature of the job or to prepare incoming committee members for the work it entails [2].

It is important to go beyond a generic exploration of the role and to understand more about the knowledge and competencies thought to be central to DMC membership. DMCs function in many different trial settings and it is reasonable to assume that the issues faced will vary with the circumstances in which they operate. Research questions, interventions under evaluation, and the research populations involved will all shape what is required of DMCs. Understanding setting-specific challenges is therefore crucial to understanding the work of different DMCs, and will promote a better understanding of both commonality and variance. One specialty where this possibility of setting-specific challenges may be explored is neonatal intensive care.

We might expect trials in the neonatal intensive care setting to involve similar issues to trials in other settings with technological and clinical similarities such as paediatric and adult intensive care, or paediatric oncology where trials also involve sick children and their families. Parekh and colleagues [13] have, however, argued that complex considerations for neonatal intensive care trials set them apart even from trials in apparently similar settings. Rather than assuming commonality, it is important to consider the potential for unique issues and challenges which might flow from the context in which trials are set.

Neonatal intensive care trials often recruit and deliver interventions to critically ill participants, at high risk of death. They are an unpredictable and variable population. Complex clinical needs which arise from neonatal physiology are not common to other patient groups. Some of the babies recruited into trials are born at full term but are injured or become sick. Some are extremely preterm, as low as 23 weeks gestation at birth, and even basic care to facilitate breathing, feeding, and pain relief can be difficult to deliver. Sick neonates can deteriorate rapidly. Severe complications can unfold at the same time as, but be unrelated to, the focus of a trial and its intervention. With families in crisis, complex social situations can underpin trial recruitment and follow-up. Treatment decisions, which can be shaped by inclusion in a trial, can have life-long effects.

With such a vulnerable population, safety is a key consideration and a DMC offers an important means to safeguard the wellbeing of trial participants. The majority of neonatal intensive care trials do establish a DMC; Perrem and colleagues [14] identified 61% of neonatal intensive care trials published in four journals as having convened a DMC.

DMC members for neonatal intensive care trials were among key informants in the BRACELET Study, which focused on issues related to death and bereavement in paediatric and neonatal intensive care trials [15]. The study included a qualitative component focusing on a group of neonatal intensive care trials with populations at a high risk of death, and considered the views of trialists, clinicians and parents associated with these trials. Within this larger framework, we were able to explore with those who were DMC members the challenges they faced in relation to trials in neonatal intensive care, and how these might differ from those in other settings. This paper describes and explores those challenges and situates them in the context of neonatal intensive care. The aim is not to guide DMC practice but through the views of those involved, to gain insights into experiences of DMC membership in this setting.

## Methods

### The BRACELET Study

The qualitative component of The BRACELET Study considered death and bereavement in the context of five neonatal intensive care trials which we refer to as the Core Trials: INIS [16][17], TOBY [18–20]), PROGRAMS[21][22], BOOST II UK [23][24] and the EXPN Feeding Study [25,26]. The BRACELET study involved interviews with bereaved parents, recruiting clinicians and trial team members associated with these trials. A full and detailed account of the complex identification and recruitment processes for the BRACELET Study has been reported [15]. Here we will focus on the subgroup of interviewees who reported having a DMC role in one of the five Core Trials or any other neonatal intensive care trial.

### Sample

Eighty-seven potential participants were identified as having a role for one or more of the five Core Trials. These included investigator, trial manager, Trial Steering Committee (TSC) member, DMC member, and statistician. While these were all considered part of a trial team for the purposes of BRACELET, DMC members and independent members of the TSC are classed as ‘independent’ of the central team.

Four individuals were found not to be eligible, one was unwell, and three were no longer contactable. Of the remaining 79 individuals, 72 were invited to take part (recruitment was staggered, and the sample was already complete before the final seven potential interviewees could be invited). Sixty of the 72 invitees agreed to take part (83% acceptance) but two could not be interviewed for logistical reasons. Hence, 58 interviews were carried out. Eighteen of these were DMC members for at least one neonatal intensive care trial. One decliner was a DMC member (95% acceptance for the DMC component).

The subset of 18 DMC interviewees included 11 men and seven women: 12 were members of the DMCs for one or more of the five Core Trials. Three interviewees were aged 41–50 years, six between 51–60 years, eight between 61–70 years, and one was over 7o years old. Eleven interviewees were university professors. Table 1 details interviewee pseudonyms and the capacity in which they had served on a DMC only, in order to minimise opportunities to identify and attribute comments to individuals and specific trials.

**Table 1:** Interviewee pseudonyms and the capacity in which they served on a DMC.

Pseudonym	Representative role on the DMC
Roger	Neonatologist
Duncan	Neonatologist
Harvey	Neonatologist
Simon	Neonatologist
Naomi	Neonatologist
Domenic	Neonatologist
Max	Neonatologist
Bill	Neonatologist
Dulcie	Statistician
Celia	Statistician
Polly	Statistician
Dexter	Statistician
Daphne	Statistician
Harriet	Lay member
Hilary	Ethics representative (clinical)
Howard	Ethics representative (clinical)
Philip	Health Services Researcher (clinical)
Austin	Health Services Researcher (clinical)

DMC members’ expertise is in demand, and several interviewees were investigators, recruiters and/or TSC/DMC members for more than one trial at the time of interview. Most were senior members of their clinical and academic community and as such had a history of involvement in landmark trials. Lay members and ethicists have important areas of expertise to contribute but are involved in DMCs less commonly. This is reflected in our sample composition. These multiple roles and experiences fed into and informed the BRACELET Study interviews and so the data presented below relate to a much wider range of neonatal intensive care trials than simply the five Core Trials.

### Interviews

Semi-structured in-depth interviews were carried out by one interviewer (CS) between June 2009 and July 2012. The interview schedules were developed by CS and DE in consultation with the wider BRACELET Study team. The interview schedules were used for the wider set of interviews for the BRACELET Study and so needed to be adaptable for different and sometimes multiple roles for neonatal intensive care trials, one of which was DMC member. The interviews included questions relating to neonatal intensive care trials generally, the specific trial(s) for which interviews had a role, and any other relevant roles they had. A core set of simple questions were built in to the interview schedule to open up discussion of key areas of interest in relation to the DMC experience which would build on earlier discussions. These questions were a starting point for an informant-led discussion to cover issues which were important to them. As each schedule differed according to the profile and trial portfolio of the interviewee in question, no single interview schedule exists, but the areas considered included exploring particular challenges; e.g. how statisticians, clinicians and others work together within the DMC; decision-making; responsibility; burden; and keeping one’s nerve.

Fourteen interviews were conducted in the workplace, four were conducted in the interviewees’ home. The interviews covered a wide range of issues in relation to the Core Trials, trials in general and the role and remit of DMCs. The subject of DMC practice was explored in detail with interviewees often branching out into new areas for discussion in the light of their own personal experience and views.

### Analysis

All interviews were recorded and transcribed. For each interview detailed, reflexive field notes were written by the interviewer (CS) which included a description of the tone and flow of the interview, and any points of significance. Emergent issues and points of importance were noted. The notes were shared with other BRACELET team members to afford them insights into the interview processes, and were extended after having interviewed the whole sample, and used by the analyst (CS) to gain an overview of the dataset.

Transcriptions were analysed using the qualitative package, Atlas.ti [27]) which allows data to be grouped according to broad overarching themes, and significant segments of text to be coded (labelled) and annotated. As analysis progressed, themes and codes were refined using a constant comparative approach [28] and the team focused in on key insights. The larger BRACELET Study is an interpretive phenomenological study wherein a phenomenon, for instance parents’ experiences of consenting to a trial, is understood through detailed accounts in relation to a given context [29]. This approach informed analysis of the subset of DMC data, especially in terms of the importance of the neonatal intensive care context in which the DMC experience is set, but the analysis of the DMC component was more descriptive and thematic than interpretive. The aim was to use views and experiences described to understand the nature of the challenging work carried out by DMCs in this setting, rather than to explore it as a phenomenon.

### Ethical approval

Research Ethics Committee (REC) approval was given for the qualitative component of the BRACELET Study by the North West Research Ethics Committee (NWREC) in April 2009 (ref no: 08/H1010/113) and in June 2009 by the Research Ethics Committee for the London School of Hygiene and Tropical Medicine (ref no: 5533), which acted as study Sponsor. A substantial amendment was approved by the NWREC in September 2010. Research and Development approval was given in all participating centres.

## Results

Discussion of the challenges faced for DMCs, were for most interviewees firmly grounded in the neonatal intensive care setting, especially for those working as clinicians. The characteristics and needs of the patient population, the clinical issues involved, and concerns to drive forward a strong and sound evidence base for care ran throughout the data. Interviewees regularly referred back to the neonatal intensive care setting, and through detailed explanations strove to make the particularities of a topic under discussion clear. Interviewees with a non-clinical background, in particular the statisticians who had a strong focus on interpretation of data, often took a broader approach in their discussions, but the neonatal intensive care setting was still a strong component in their interviews.

Dulcie, a statistician, was clear that DMCs were important in this setting:

*[S]ome DMCs are almost kind of window dressing*. *There are some trials that quite frankly you don’t need them for*. *[In neonatal intensive care trials you need]* …. *the reassurance [that]* … *that protective mechanism is in place*.

Understanding how neonatal intensive care is different from other settings was seen by interviewees as a crucial component of DMC discussions. Philip explained how it is important when convening a DMC to ensure a good understanding of trials in this field, that it is crucial to *“make sure we’ve got an experienced member in the DMC who has sat on perinatal trials*.*”* This was not simply a reference to familiarity with neonatal intensive care and its relation to perinatal care more generally, but a deep understanding of the significance of a complex array of clinical features, interventions, responses and how these manifest within trial data. Another interviewee, Roger, who had served on multiple neonatal DMCs, suggested that there is a shared and understood way of working in this setting. He described a neonatal intensive care DMC where the chair came from outside the specialty.

*It felt different to some of the other committees*, *partly because it was chaired by a non-neonatologist*.

He felt that the chair worked with priorities grounded in a different clinical setting. He explained that the difference a small number of days gestation could make to outcome might not be clear to those unfamiliar with neonatal intensive care. In a committee guided by a chair from outside the specialty, priorities such as exposure were initially at the fore. When Roger said: “*[the Chair] was worried about* … *what was being given*, *rather than necessarily the outcome”* he was describing not only a different knowledge base but also an approach to DMC deliberations driven by concerns from a different field.

This strong sense that DMC work was driven and shaped by the priorities of neonatal intensive care trials, which closely map onto the priorities of neonatal intensive care, ran throughout the interviews. Harriet, a lay DMC member, observed this connection in the approach of a neonatologist on her committee. She said: *“[Y]ou could feel she was* …, *straight out of the clinic*, *into the data monitoring*, *and [straight back to] her day-to-day work in dealing with these sorts of circumstances*.*”* The connection was brought into focus by interviewees’ responses to two key outcomes, death and severe disability, which loom large in neonatal intensive care research and practice. They dominated the interview discussions and were a major interlinked theme in the analysis. We therefore focus here on how DMC members responded to death and severe disability as key features of neonatal intensive care practice and research, and how this was mirrored in the practice and thinking of the DMC members.

### Death and severe disability in the practice of DMCs for neonatal intensive care trials

That the topic of death was so prominent in the interviews was inevitable given the BRACELET Study focus on death and bereavement in neonatal intensive care trials. The topic was highly salient to discussions of care, research, and DMC practice. The topic of severe disability was introduced by the first interviewees who presented it is as equally as important a consideration as death in this context. Interviewees commonly mentioned that death and severe disability are often considered in close proximity as outcomes in neonatal intensive care trials. When asked about the equivalence of death and severe disability in the neonatal context, Arthur spoke for many when he said *“they’re not the same but they are both catastrophic*.*”*

Many babies in intensive care are at high risk of both outcomes and the neonatologists on the DMCs regularly deal with death and disability in tandem. They are involved with end of life decisions and processes, and there was no sense of inurement to the subject or the experiences involved. Harvey explained that “*It’s something that all neonatologists take very*, *very seriously and it is something that they always remember*.*”* The neonatologists also run follow up clinics for children discharged from neonatal intensive care. They see the consequences of preterm and traumatic birth, the impact of interventions, and witness the full disability spectrum first hand. Their views are important in informing DMC discussions.

Having witnessed the process and impact of death and bereavement so closely, it was notable that none of the clinicians, or indeed any of the 18 interviewees, stated that their aim was to always try to avoid death. Rather there was an acceptance that death was an inevitable feature of neonatal intensive care. There was not, however, evidence of an equivalent acceptance of severe disability. Simon spoke of the possible ramifications of an intervention tipping a fragile balance to survival, using the example of hypoxic ischemic encephalopathy. This condition arises from oxygen deprivation at birth. Until relatively recently, there was no way to prevent brain damage from developing in the days that follow.

*[[I]f you provide respiratory support at the right time for the right length of time you will get babies who will survive who* … *almost certainly wouldn’t have done otherwise*. *And though they do survive they don’t make any neurological progress* … *[Y]ou end up with a broken family and a dead handicapped baby at the age of ten*.*”*

Simon’s comments highlight the delicate situation in this setting where an intervention may tip the balance to survival and change multiple lives in all sorts of ways. Several interviewees in the wider study expressed concern at the possibility of a trial intervention leading to severe disability. Roger said “*You are terrified of doing harm*.*”* Views of *t*he nature of harm in the context of a neonatal intensive care trial will be explored below.

Interviewees commonly explained that the importance of death and severe disability in this setting means that neonatal intensive care trials may use composite outcomes. While there are also statistical and resourcing issues in favour of the use of composite outcomes, this approach fits well with concerns in the setting. When the incidence of one or both of these important outcomes separately is very low, using a composite can allow a trial to recruit a smaller target population than taking either as separate primary outcomes. This is particularly the case if the effects of the intervention on these outcomes are likely to go in the same direction—that is the intervention may result in a reduction in both death and severe disability. Some trials however, may anticipate the possibility that an intervention may result in a reduction in death but an increase in severe disability (so-called competing risks). Even in such cases, a composite outcome means that both components can be fully considered in the interpretation of effects.

The use of death and severe disability as a composite outcome in neonatal intensive care trials was such a major part of early interviews that it was incorporated into the interview schedule and discussed with all subsequent interviewees.

### Composite outcomes of death and severe disability

Commonly interviewees explained how the use of death and severe disability in neonatal intensive care trials is different from using the same composite pairing in other clinical settings.

Philip said *“In neonatal intensive care almost all the deaths are early on* … *there’s almost nothing later on”*. This means that accruing trial mortality data in this setting can be quite straightforward. Data can be collected from a neonatal intensive care unit not long after a trial participant was enrolled and the mortality dataset might be complete relatively shortly after recruitment closes. This is in contrast to trials in specialties where mortality may be assessed at a much greater distance from recruitment, and where the completeness of a dataset will in part depend on the success of strategies to ascertain deaths. The timepoint selected for recording survival and mortality is also important: Philip illustrated this with the example of cancer trials: *“… the longer the [follow-up period] the more deaths"* and this needs to be taken into account in interpreting the data.

Assessing neurological impairment, or disability, is by contrast, likely to be much more complicated for neonatal trials than in other settings. In trials in specialties which might also measure degree of impairment, such as adult stroke or trauma, initial deficits can be assessed relatively quickly: can the person speak at testing, follow instructions, use fine motor skills? Can they walk? For neonatal trials these outcomes cannot be measured until participants have reached or passed standard developmental milestones. Philip summed up the implications for neonatal trials:

…‥ *disability has to wait until two years of age to assess*. … *You have to wait a long time before* …. *[you can] offset it*, *with the deaths*. … *[I]t’s a unique situation*.

Daphne explained why having access to information about both death and severe disability is important. A trial for which she had served as a statistician had faced exactly this sort of temporal split in data collection:

*[O]ne of the things we’d gone into the trial for initially* … *was to say death isn’t the only thing*. *We need to also be looking at severe disability*, *because of the risk that what you’re doing is salvaging babies who are going to have unhealthy quality of life later*. *So it was always up front that this was a composite outcome of death and severe disability*. … *[You had an] interim period where you didn’t have enough numbers coming through to be able to see whether* … *the numbers who were dying and the numbers who were going to be severely disabled were going to balance out*. *And that was quite a tense time on the data monitoring committee*.

**Fig 1:**
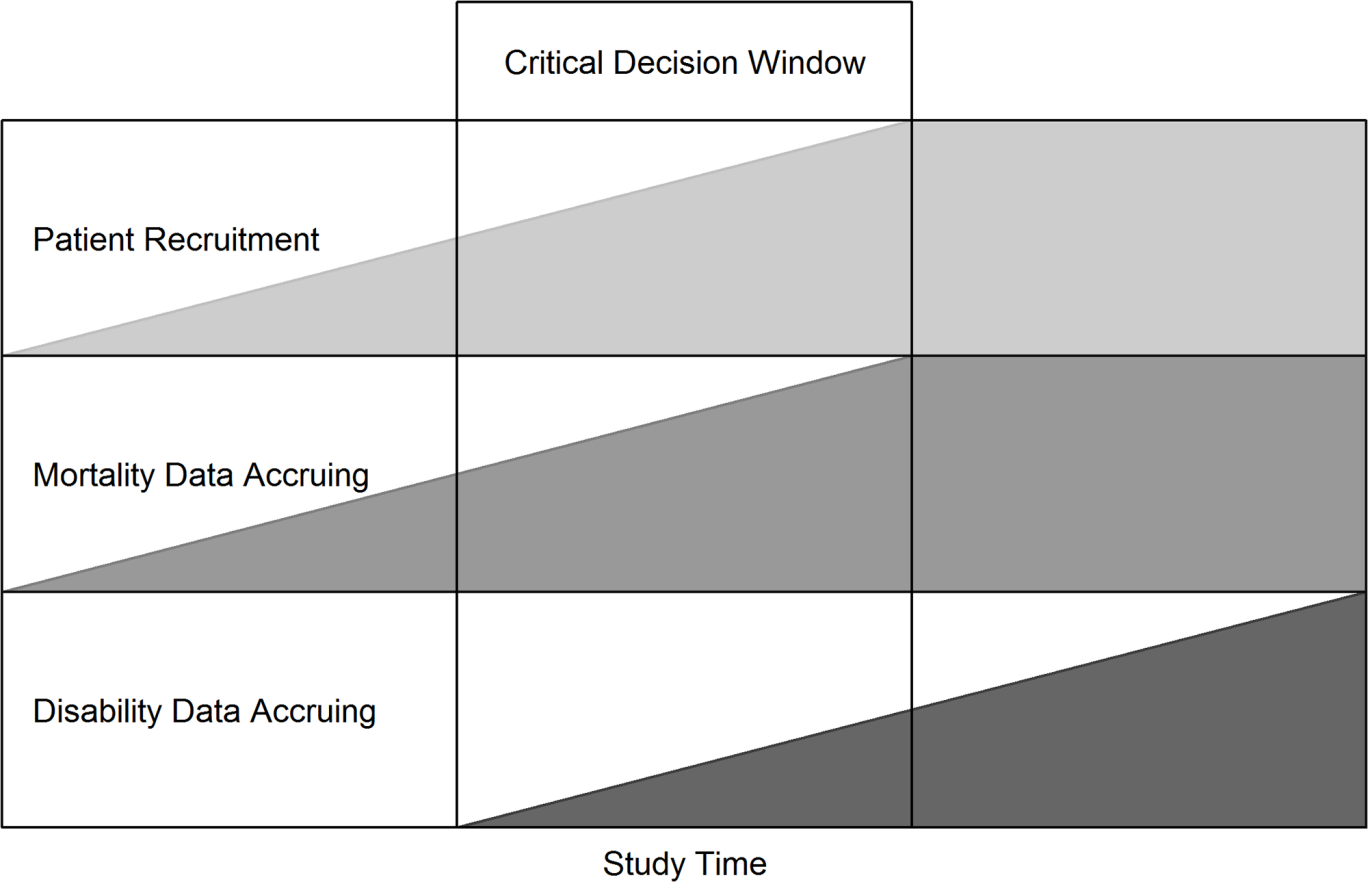
Critical decision window for DMCs.

Fig 1 shows how this situation might be manifest in a simplified hypothetical trial. The figure shows the critical decision window when it is possible for a DMC to decide on their recommendations to a TSC about continuing, modifying or stopping a trial, but in which the composition of the types of data available to inform those decisions will continually change.

Within this larger window is the “interim period” described by Dulcie, when mortality data are accruing but disability data are limited or pending, that interviewees described as particularly challenging. If, at interim analysis, trends in mortality seem to be appearing, the DMC must work through the implications of these data. In the interviews there was a lot of discussion of the need to be aware of “fluctuating data” (Austin), effects which appear and disappear, and the importance of not being distracted by incomplete, weak or problematic evidence in this situation. Dulcie, referred to the importance of not *“stopping on a random high”* because *“the first point at which you think “Oh dear*, *it’s gone over the threshold” is almost bound to be a blip”*.

*[Y]ou’ve got to be very careful that a result you see early on doesn’t stop the trial when actually you then haven’t got a proper handle on [*things]. *So in terms of data monitoring*, *you’ve got to* … *give the data long enough to make sure that you’ve got a handle on that balance*.

Interviewees spoke of a number of tensions which come to the fore when a DMC looks at interim data in this situation and must recommend to a TSC whether to continue or to stop recruitment. Hugo pointed to the difficulty in waiting for morbidity/disability data to accrue.

*The heart of the data monitoring committee is the question about whether it is ethical to continue with the trial or not*. *And you can only do that on the basis of the thing that you have at the time and obviously [in this setting] it does tend to be mortality before morbidity*.

The challenges involved were not minimised and were evident in the commonly used image of *“losing”* or *“holding your nerve”*. Domenic said:

*I’ve seen trials stopped for no good reason because the DMEC took an over-nice view of*, *of what they were supposed to be doing*. *[In] the [XXX] Trial* … *the DMEC stopped it after*, *I think it was two* [adverse events] *and the statistics did not hold that up*. *They just lost their nerve*.

Harriet described the human emotions which make holding your nerve a challenge:

*You certainly have to hold your nerve*. *You* [have to keep] *your feelings where they belong*. *In other words*, *they must underpin everything you do*, *but it’s about reason overcoming emotion*. *You have to feel the emotion*, *but you have to hang on to your reason all the time*. *You mustn’t let it sway you*.

We consider below the issues faced when these two options, continuing or stopping, are under consideration, and the elements which might contribute to holding and losing nerve.

#### The choice to recommend continuing or stopping recruitment

When DMCs recommend continuing with recruitment as planned, it is most usually the case that this reflects a situation in which there are no concerns. Austin commented: *“nine out of ten trials are straightforward*. *It’s the tenth trial where it’s difficult*.*”* One important consideration commonly raised by interviewees, as in Philip’s comment below, was the need for trials to produce sufficiently robust evidence which people will use in practice. Philip referred to a “lesson” from a trial outside the neonatal intensive care setting evaluating streptokinase for myocardial infarction:

*You know*, *lots of people argued that the ISIS trial*. *which was set up with*, *I don’t know how many thousands of*, *of people in it*, *it was unethical [because] we already knew the answer*. *Well we did but nobody had changed their clinical practice*, *nobody was using it and in the context of nobody using it*, *it was entirely ethical to do another* …. *trial which was big enough to*, *by itself*, *to give a clear answer*, *despite all the previous trials having been done since the nineteen seventies*.

Determining whether the data accumulated by a trial meets the level of evidence needed can be difficult, especially when stakes are as high as they are in this setting. The interviewees were very much aware of the issue of perceived effects fluctuating over time (as Dulcie mentioned above) and told a number of cautionary tales about trials in which a DMC saw a trend emerging only to see it disappear over time. One of the statisticians, Celia, argued that there can be other ways in which data can be misleading and warned against temptation in committees with a mixed skill set in the membership, to over-work the data at this tense time:

*Obviously the other members of the data monitoring committee are not going to be statisticians and* … *actually there is a lot of explanation required*, *you know to understand* … *the whole concept of*, *you know*, *the more times you look at the data the more likely you are to find a P value smaller than*.*05 and so that you have to actually take account of the number of times you look to decide on what criteria you use for stopping the trial*.

One of the troublesome areas for interviewees was deciding whether a threshold had been crossed in terms of the evidence needed to support or change clinical practice or whether a trial should push on with recruitment. Several interviewees referred to the value of pre-agreed stopping guidlines but discussions suggested that how these might be used would vary. Howard said: *“some DMCs have really very difficult decisions to make*. *But no matter what has been set out at the beginning*, *they still have sort of grey areas and areas of difficulty*, *and areas of debate*.*”* Outside of his formal interview, one of the interviewees argued that there would be no need for a DMC if things were as simple as stopping a trial as soon as predefined conditions had been met. Deciding what the guidelines should be, and subsequently whether or not to action them, was seen as part of a much wider discussion than just about death and severe disability data.

It was clear from the interviews that a lot of DMC deliberations focus on the human issues as well as on the statistical data, that the two are held in tension, and central to this is the issue of harm. Again the statisticians made observations about how their colleagues react to the interplay of data and human cost. Celia commented that in a protocol there may be symmetrical stopping rules for benefit and harm. She did not feel that this symmetry was reflected in DMC practice:

*[D]ata monitoring committees* … *do not have symmetrical thought processes*. *…[They] are very concerned about harm*. … *I was on data monitoring committee* … *and they were willing to allow a trial to continue for much longer if they thought the [intervention] was potentially beneficial than they would if there was any suggestion of harm*. *Data monitoring committees are very*, *very concerned about continuing a trial if they think the treatment is actually causing harm*. … *[and] won’t use statistical stopping rules for harm that they will for benefit…*. *[*T*]heir nerves are strained much more if they see harm than if they’re seeing benefit*.

Dexter however saw the issue of symmetry differently and saw utilisation of different thresholds as appropriate:

*[I]n the data monitoring context the guidelines for stopping a trial early for harm are often different from those for stopping for benefit*. *So that the amount of evidence you need of harm is not as great to stop a trial*, *or to recommend stopping a trial*, *as the amount of evidence you need to recommend stopping for benefit*. *There’s an asymmetry*, *which is often built in to a protocol*.

The issue of the possibility of harm was clearly prescient for many of the interviewees. Roger, a neonatologist, made a comment about the difficulty of balancing the need to protect individuals and the need for robust evidence to guide practice.

*“You’re terrified of doing harm and of being accused of not stopping it in time*, *but equally you don’t want to throw away the only opportunity you may well have of answering that question and benefiting children in the future*.*”*

Dulcie had experienced exactly this situation in a trial for which interim results were suggesting *“that the standard was better than the new*.*”*

*My first experience of data monitoring was actually a trial [where the new treatment]*‥*did harm*. *…*. *[I] went along to this meeting and there was a barney [an argument] going on*. *…*. *[A DMC member] said “Actually it’s unethical to carry on randomising people to find out how bad this treatment is*.*”* … *And some people said “No*, *we should carry on to be sure of this”*

In neonatal intensive care the personal stakes can be very high and where death and severe disability are likely outcomes, and continuing a trial to consolidate evidence may have major consequences for some. Philip focused in on just this challenge, describing a difficulty that would arise with a just significant result which *“may not persuade people to change their practice*.*”* In such a situation he said:

“*it may be justifiable—and I use the term “may be” [because] these are awful decisions—to go on to accumulate more deaths in order to change practice* … *for the myriad of babies out there for years to come who may benefit from that intervention*. … *You are essentially balancing the need to get reliable strong data for the future against that group who would basically now come into the trial just to strengthen the finding*.*”*

Picking up this thread in a later interview with Austin, the interviewer focused on the point at which a DMC is deciding whether or not to continue, and asked “does the fate of the next person randomised … recede from your thinking or is it … prominent?” Austin replied:

*I think the individual ethic and the sort of collective ethic* … *coincide*. … *[Yo]u want the results to be certain results*, *for both the future individuals being randomised and future people who* … *will receive the treatment after the trial*. … *[I]f you’re not sure that the result will convince practice in the future*, *then you’re not sure if it’s*, *if it’s appropriate for the next patient who might be randomised*. … *[T]he point where it’s clear for the next person who might enrol in the trial*, *it’s also clear for the next person who will be out with the trial*.

A decision to continue can clearly be morally complex and is not only a trade-off between evidence and participant/patient needs. Howard explained how there are real life practical considerations which can also feed into DMC deliberations. He was mindful of how a decision to stop a trial could even change things for patients outside the trial and not yet born. In the example he described, the intervention was available only within the trial and only to those allocated to the intervention arm. He set the scene:

*We’re starting to see a beneficial effect of [the intervention]*. *We haven’t met the criteria to say it is clear that there is an advantage to this therapy*.

The DMC had to decide whether to act on this weak sign of benefit, or continue. Howard said their decision to continue related in part to the availability of the intervention in the UK. The trial used all available UK facilities for that intervention. If they stopped and recommended that the intervention be used at that point, they would remove the restriction of access only via the trial intervention arm, but no additional babies could be treated. Stopping and opening up the intervention would have changed who, but not how many, could access the treatment. The decision was made to continue with the trial to ensure robust and reliable data.

The interviews suggested that in making these decisions, DMC members feel a strong sense of responsibility which comes from an awareness that their recommendations will impact upon people’s lives, and from reflexivity, an understanding that their own and others’ value judgements are part of the decision-making process. Responsibility and reflexivity as dimensions of the DMC role are explored further below.

#### Responsibility and reflexivity

From data presented above it is clear that DMC members felt a sense of responsibility for the quality of scientific evidence, for the potential to change practice, and above all for the variety of ways a decision might impact upon people’s lives. In some interviews there was discussion of a collective sense of responsibility, the DMC working together in their deliberations, but there was also a strong feeling of personal responsibility. These two dimensions were articulated most keenly by the lay DMC member, referring to the *“weight of responsibility”* she felt.

Harriet explained how the DMC worked together in a *“pooled”* way to contribute their expertise and understanding and to share responsibility.

*We felt we had to have*, *as it were*, *a certain sort of detachment from what we were doing*. *In other words it mustn’t be personal*, *and yet everything we do is based on the personal*. *We have always to bear in mind this balance of*, *do we go on*? *Do we stop*? *How is it panning out*? *What is the effect of continuing*? *In other words the very broad issues of being involved in such a responsible committee*. … *You’ve got the lives of all these hundreds of babies*, *and the lives of all these families that you’re dealing with*, *under your scrutiny*, *and everybody’s contribution* … *has a bearing on what we’re trying to do*. … *You’re focused on the task in hand*, *and you sort of subsume your own personal feelings*, *I suppose*, *and use all the attributes you’ve got in order to*, *to give the best that you can to what you’re doing*.

Harriet’s reference to *“all these hundreds of babies”* pulls together all the different groups the interviewees in this study considered in their discussions, babies within and outwith a trial, babies exposed to risk and those excluded from advantages, the masses who will be cared for using information from a trial, or those who will not benefit if a trial fails to provide evidence for a change in practice. When she talks of the lives of these families she conveys a sense of the magnitude of the task in hand.

For Hilary, the personal and individual sense of responsibility she felt became clear over the course of the interview. When she first agreed to take on a DMC role, she did not do so lightly and had read about the work of DMCs to prepare herself. Even so, she found the experience more challenging than she expected. She said:

*I would never have* … *realised*, *had I not done it*, *quite what role the DMC plays*. … *I think you have to be in that situation to realise what it feels like to have access to that information; for other people to be depending on you to make that judgement*.

A trial she worked with was stopped early, a decision Hilary described as *“a fine call”*. Having previously agreed to continue with recruitment, things changed at a meeting where the decision was made to recommend to the TSC that the trial should be stopped immediately.

*[I]t couldn’t wait*, *you know because it was so clear*. *[W]e had these years of looking at this stuff and*, *and you know saying*, *"Now is it right*? *Are we actually sure it’s right to continue*?*" and then suddenly to have this point where you’re saying*, *"No*, *we should not be continuing*.*" You know my heart’s going out to all these people who had gone before*, *where we*, *as the DMC*, *had said yes*, *keep going*, … *and that has really troubled me*. *I mean*, *it was done absolutely in good faith*. *We could not have done anything different but it*, *it really I think made me feel that all my intuitive worries about taking part in this study had not been ill founded*. … *[I]t was all of that*, *that I was thinking of*, *you know*, *what would I feel like if I had been instrumental in continuing this*? *You know*, *would I have the lives of these children on my conscience*?

Hilary had struggled somewhat after the close of the trial and said she would have liked further opportunities to discuss what had happened with other DMC members and to *“debrief”*. She referred to *“the onerous responsibility on a DMC”* and said that she did not realise the extent to which *“you personally feel that responsibility until after the event”*.

Interviewees expressed their awareness of the fact that some of the decisions they made on DMCs and for which they took responsibility, were value judgements which would not necessarily be shared by others. Naomi described a situation in which a TSC rejected a DMC decision. She said:

*I learned a lot from that experience because it made me realise that a DMC can*, *and on occasions should*, *be challenged and can be challenged successfully*. … *Data Monitoring Committees after all are only a small group of people …with a particular set of opinions as opposed to another group of people with another set of opinions*.

Several other interviewees mentioned situations where a TSC has rejected a DMC recommendation to stop a trial, pointing to this as evidence of different ways of viewing a situation and interpreting data. Dulcie spoke of the importance of reflexivity, of understanding the values that are informing the decisions that are made, and how they have to be “*factored into your thinking”*. She argued that those with key responsibilities need to discuss what should be valued. Any differences between members of the DMC, and differences between the DMC and the TSC, need to be made clear and worked through. She felt that if this could happen before a trial starts to recruit and before a DMC starts to work as a closed group, there is the possibility of including a wider group of contributors, including lay TSC members, in those influential discussions.

Interviewees were also aware that their decisions rest on values which are highly personal and may not be shared by some of those whom they most profoundly and directly affect. In thinking about possible differences between DMC and parental perspectives, Philip made a connection between data, evidence, power and values. He first focused on DMC processes, referring back to the temporal split discussed at the beginning of this paper. He said:

*[Y]ou may be sitting on a difference in mortality waiting to get some supporting evidence that you aren’t getting an increase in severe disability which may offset the difference in mortality*. … *[But] what do we mean by offset*, *whose judgement is that*? *If you told the parents that* … *there’s no difference between the groups [overall] but one has a* … *[relatively higher] risk of death and the other one has a [relatively higher] risk of disability…… Some will say*, *“I don’t care if my baby’s disabled*, *I want my baby to survive” and others will say*, *“I don’t want a severely disabled baby*, *it’s not fair on the child”* … *[T]hese are very difficult philosophical decisions*.*”*

Simon’s comment reported earlier, referring to *“a broken family and a dead handicapped baby by the age of ten”* is a case in point. For him it was a heartfelt view of severe disability which informed his practice but is not one that all parents of disabled children would share. Dulcie commented that people *“value those things very differently”* and pointed to the challenge this raises for DMCs.

*[H]ow you weigh those things up in a data monitoring context* … *when actually there’d be a heterogeneity of opinions among parents* …. *there is no sort of one right answer*.

## Discussion

The testimonies of the DMC members represented here combine to explain different aspects of their practice, deliberations and personal experiences. For those not privy to DMC meetings, this account may clarify some aspects of the work involved. It explores some of the common images available in the literature on DMCs, of burden, responsibility, courage and the knowledge base needed to carry out the role, and considers these in relation to neonatal intensive care trials. It highlights some of the factors which seem to be specific to this particular context, in particular the effect of the availability of different types and levels of data in the critical decisional window available to DMCs for neonatal intensive care trials. Other such research is necessary to explore key issues in different clinical specialties. Until such research is carried out it will be difficult to know what is generic, common to many settings, and what are the challenges, scientific, ethical and personal, that DMCs have to meet when they work with trials which are subject to different specialty-specific influences.

These data tell us something about the scope of the DMC role and shed some light on issues which might be specific to neonatal intensive care trials. We identified the temporal split between death and severe disability which can be inevitable in this setting when trials use composite outcomes. As the stakes are high in neonatal intensive care, with the death of an infant having lifelong implications for families, and severe disability set up in the neonatal period having lifelong implications for children and their families, this temporal split challenges DMCs whenever it arises.

It was suggested by some interviewees that this temporal shift may feature less in the future as neonatal intensive care trials focus on interventions which are less likely to affect mortality. Parekh and colleagues [13] argue that major advances in care have increased survival such that it is the condition in which survivors survive that is now the priority. As a consequence, trials may be driven by a focus on the impact of an intervention on morbidity and long term neurodevelopment with mortality as a lesser focus. It is not, however, a simple refocusing. Mortality can be an unexpected consequence of an intervention; one of the Core Trials in the BRACELET Study was stopped on the recommendation of the DMC because of a difference in mortality, with all active trial participants in one of the intervention arms immediately moved to a new treatment modality. Evaluating an intervention not designed to improve mortality will still be taking place with a population at high risk of death. This will always raise methodological questions for trial design and evaluation and bring death and severe disability into close proximity. It raises social and ethical concerns when trials involve an intervention at the start of life and where consequences for participants and their families may be life-limiting or life-long. As trial methodology changes and develops there will be new challenges ahead[30][31].

If DMC members are to be adequately prepared to take up their role and meet these challenges, then training is appropriate. Zuckerman and colleagues [2] argue for specialty-specific training for DMC members in the context of allergies and infectious diseases. Given our findings we would also suggest that training in relation to DMC membership for neonatal intensive care is needed. Other specialties could make a similar case. Specialty-specific training could be provided along with training on a core of general issues encountered by DMCs, for instance the range of statistical stopping guidance they may use, and the role of the DMC as advisory to the TSC. This can, however, only happen with careful investigation of what a specialty-specific DMC role entails across the range of circumstances that might pertain for a given setting.

It is however important not to see training as the only way forward. This work has revealed how careful these DMC members felt they had to be in their deliberation, how their collective lay and professional skills input to the challenges of interpreting data and the workings of the group. They valued the neonatal intensive care experience that members fed into the DMC, and prior experiences of DMC membership was important. The collective skills were clearly highly valued but for these interviewees were largely a product of experience, and not prior training. Almost all of the interviewees had served on more than one DMC and most were in the later part of their careers. Most drew on many years of experience in their particular field. One possible way to prepare those planning to take on a role as DMC member, to help them to gain insights into the challenges involved, is to open up DMC meetings to observation, in confidence, with mentorship arrangements helping novice members to learn from experienced colleagues, and to grow into the role.

Key images mentioned in the literature were burden and responsibility. While it was clear that the DMC members interviewed here felt a keen sense of responsibility, there was little sense that they felt burdened by the responsibilities of the decisions that they had and might make. This is despite being involved in trials which had involved difficult decisions to recommend to stop or continue, as detailed in our findings. Harriet referred to “*a certain sort of detachment”* while also maintaining an *“awareness of lives of hundreds of babies”* and “*hundreds of families”* and clearly this balance is important. The account from Hilary however, indicates that there is the potential for the DMC role to become onerous and burdensome. She explained how she had struggled with her sense of personal responsibility and would have liked to meet again with other DMC members to debrief. This is not a simple issue to tackle. It may be that training may prepare people for the potential for difficulties should their committee have to face, and make, challenging decisions, or deal with situations such as harm to participants. It is important to find out what preparation or support might be useful and what is feasible even if this is needed for a minority of members. This is especially important to help new members transition into their role, and for DMCs choosing to include lay people in their membership because of their associations with a particular patient group.

The issues highlighted here, the scientific, ethical, personal and setting specific challenges that have been described, and the evolving nature of clinical care and trial methodology, confirm the demanding nature of DMC membership in this setting. One of the interviewees summed up the different demands of the role of DMC membership, saying:

*[You need] people who* … *understand the way that data fluctuates and that have the ability to balance the interests of the participants and science* … *[and know] what sorts of results would actually have an impact*.*”*

Austin

His words capture the fact that the role requires not only a key set of skills but also preparedness to take on the potentially *“onerous responsibility”* that weighed heavily upon Hilary. If DMC members of the future are to be well equipped to take on these responsibilities, they will be well served by research and training which will further elucidate and support them in the challenges they face. Research and training will, however, need to keep pace with a changing complex mix of methodological, technological, clinical, ethical and social work.

### Conclusion

In a recent review article, DeMets and Ellenberg [32] note that “*DMCs have to be prepared for the unexpected*, *relying in large part on their experience and collective wisdom”*. This view is reflected in the BRACELET Study interviews. DMC members brought an empathetic dimension to discussion of death and severe disability along with a strong sense of real world issues, and so went beyond a theoretical or academic treatment of the topic. Some drew on their experiences as parents and grandparents as well as professional experiences. All saw these topics as being of central importance in the work of the DMCs, as common outcome measures in neonatal intensive care trials but also as central weighty issues of human and clinical significance.

The work of members of neonatal intensive care trial DMCs is particularly complex in that they consider death and disability in tandem, at the population level but also for the individuals they are duty bound to protect. Members are selected for key skills and reputation and are aware that they are trusted to deal with the onerous challenges of assessing, interpreting and monitoring data to support the good and safe conduct of clinical trials. DMC members identified dealing with composite outcomes of death and severe disability as a key and demanding issue. This, and the wider issues faced by DMCs, requires members with a range of specialist skills and insights, who will work as a collective but are also prepared to shoulder a sense of responsibility for decisions which they acknowledge have far-reaching consequences. DeMets and colleagues [33] argue that DMCs *“should consider ahead of time the possibility of unexpectedly harmful results*, *and should document appropriately the statistical guidelines”*, but recognise that the decision-making processes are complex. This view is mirrored in the BRACELET Study interviews where pre-agreed guidelines were seen to be useful, but the role of discussion within committees was seen as crucial and the presence of value judgements in the choices and recommendations they make were discussed. The interviewees were in no doubt that their decisions affect evidence and affect lives. The possibility that others who bear the costs of those decisions may not share the values of DMC members was acknowledged. DMC members have to bear responsibility for all of these things and in doing so felt that they have to hold their nerve.

DeMets and Ellenberg [32] state: *“The evolution of DMCs continues as the art and science of clinical trial performance itself evolves”*. The work of DMC is complex and multifaceted and will change as new clinical and scientific challenges in different fields emerge and as trial designs develop. Research is needed not only to understand any evolution which may take place, but also to foster the growth and development of the broad and possibly setting specific training needed to support DMCs in their responsible roles.

## Supporting information

S1 Appendix BRACELET Study info booklet 160418. (DOCX)
