# Supplementary material for: "You have to keep your nerve on a DMC." Challenges for Data Monitoring Committees in neonatal intensive care trials: Qualitative accounts from the BRACELET Study
Source: PLoS One. 2018 Jul 26;13(7):e0201037. doi: 10.1371/journal.pone.0201037 (PMC6062057; doi:10.1371/journal.pone.0201037)
Supplement: S1 Appendix — (DOCX) [file pone.0201037.s001.docx]

**Information booklet**

**Trial Team Members and Recruiting Clinicians**

**Version 1, 17/11/2008**

We would like to invite you to take part in the BRACELET Study. Before you decide it is important to understand why this research is being done and what it would involve for you. Please take some time to read this information carefully. Some further information can also be found on our website at **www.bracelet-study.org.uk**

**What is the purpose of the BRACELET Study?**

The BRACELET Study was designed to improve how clinical trials are run in neonatal intensive care. A first step was to determine the mortality rates for such trials; we now know from our Phase 1 survey that this is around 20%. This is not surprising as these trials involve very sick populations. What is surprising is how little we know of the experiences surrounding bereavement in this context; practice and policies, whether at the trial-level or set within individual clinical centres, have little empirical foundation.

In recent years there has been a rise in research involving parents of babies enrolled in a trial. This has led to important changes in how trials are run. Bereaved parents have, however, rarely been included. Some studies have included recruiting clinicians but there is still much work to be done with this group given the complexity of many of the issues raised for clinician-researchers. Very little research has been carried out with those who design, conduct and evaluate clinical trials. The BRACELET Study involves interviews with all three of these groups to allow us to consider bereavement and clinical trials from multiple perspectives.

**How will the BRACELET Study be carried out?**

Phase I of the Bracelet Study determined which UK trials were appropriate for study, and which clinical centres would offer the best opportunity to access potential interviewees. Four trials and six NICUs are taking part. To preserve confidentiality the NICUs will not be identified. The four “**Core Trials”** are:

- **INIS** (Non-specific intravenous immunoglobulin therapy for suspected or proven neonatal sepsis)
- **TOBY** (Whole body hypothermia for the treatment of perinatal asphyxial encephalopathy)
- **PROGRAMS** (Prophylactic Granulocyte-Macrophage colony-stimulating factor (GM-CSF) to reduce sepsis in preterm neonates)
- **ExPN (Extremely Preterm Nutrition) Feeding Study** (Improving post-natal head growth in very preterm infants: a randomised controlled trial of hyperalimentation)

We will carry out:

- 25 interviews with Core Trial team members
- 25 interviews with neonatologists who have recruited to a Core trial
- 30 interviews with bereaved parents of babies enrolled in a Core Trial

Taking account of the opinions of these groups of people will allow us to consider different experiences and points of view. These data will help to guide the design of future trials so that they can be sensitive to a range of needs, opinions and expectations.

**Why have I been invited to take part?**

We are inviting you to join the BRACELET Study because of your particular role within one or more of the four Core Trials.

**What will I have to do if I take part?**

There is a reply slip with this information booklet for you to let us know whether or not you would like to participate in the BRACELET Study. If you do decide to take part you should return the reply slip to Claire Snowdon, the lead researcher, in the prepaid envelope provided. On receiving your reply slip, Claire will contact you to arrange a time and place to meet you to carry out an interview for the study. Wherever possible we conduct interviews face-to-face but if you prefer a telephone interview then this can be arranged.

There is no set time length for interviews but they often take around an hour. When she visits, Claire will go over the study information to check that you are happy to go ahead. You can still change your mind at this stage as it is important that all participants feel at ease with being involved. You will be asked for permission to record the interview and, if you agree, to sign a consent form.

During the interview Claire will ask for your views on a number of issues related to the conduct of clinical research in neonatal intensive care. She will ask for your recommendations for bereavement-related practices. She will also be interested to hear about your experiences of this aspect of clinical trials and anything else that you feel is important. If there is anything that you do not want to talk about, or questions that you do not want to answer, that is not a problem. You can stop the interview at any point, either for a break or to end early if you wish. Claire will leave you a short questionnaire which asks what you thought about the interview.

**What are the possible risks and benefits of taking part?**

We appreciate that we are asking you to consider talking to us about a potentially sensitive subject and that this might be difficult for some people. We are grateful to everyone who takes part and we hope that it is an interesting and positive experience. There is more information about interviews on our website, including short accounts by two neonatologists who took part in our earlier research. You can follow the web link ***Taking part in an interview.*** Our aim is to benefit the broader neonatal community by helping to improve how neonatal clinical trials are run in the future.

**Do I have to take part?**

No, it is entirely up to you whether or not you participate. As the number of individuals who are eligible for interview is limited, we do hope that you will consider joining the study.

If you choose not to take part, it would be very helpful if you would still let us know using the study reply slip so we can see that our letters are reaching people. This will also allow us, where possible, to send out further invitations to other potential interviewees.

If you decline to be interviewed now, but decide at a later date that you would like to take part, you can contact us by telephone, email or through our website and we will be happy to arrange an interview.

**Will my taking part be kept confidential?**

We always keep the details of research participants strictly confidential. Any information that we use would have your name and affiliation removed.

We should point out, however, that it may be possible for others who are familiar with the field to make a guess at the identity of some of those involved in the study, especially those with very specific roles within a trial. If you have a very specialised role in one of the Core Trials, you should only agree to participate if this is acceptable to you.

If you do agree to be interviewed you will be asked to permit a recording to be made. The recording will be transcribed by a trusted transcription service and then encrypted so that no-one else can read it. A copy will be kept by Claire Snowdon at the Centre for Family Research, and another by Diana Elbourne at the London School of Hygiene and Tropical Medicine. We will also each keep a copy of the transcript of your interview with all identifying details removed.

Our procedures for handling and storing the information for the BRACELET Study will comply with the Data Protection Act 1998.

**Is there any other way that I can help?**

It would be very helpful if you would fill in the additional brief questions on the reply slip. If you take part in an interview you will also be asked to complete a short questionnaire about the interview process. These questionnaire data will be used to audit the processes involved, as well as contributing to a methodological element of the study which considers the conduct of research in this sensitive area.

**What will be done with the results of the research?**

We anticipate that the data will form the basis of a number of papers for publication. A monograph produced and published by the funders will be available on the HTA website as a free download, with an executive summary also downloadable. A number of additional papers will be published in journals.

The BRACELET Study website includes examples of publications from earlier studies so that you can see the type of material that we produce. You can follow the web link ***Previous publications*** to Pubmed abstracts, or link from the accounts of previous interviewees in the section ***Taking part in an interview.***

We will post a summary of the results when they are available and links to details of our papers on the study website where possible. If you do take part in an interview one of the study questionnaires includes a box to tick if you would like to be sent a summary of the results. You should be aware, however, that we do not expect to report our results until late in 2010 at the earliest.

**Who is funding the research?**

The BRACELET Study is funded by the National Institute for Health Research, Health Technology Assessment Programme. The investigators are Dr Claire Snowdon (London School of Hygiene & Tropical Medicine and University of Cambridge), Professor Diana Elbourne (London School of Hygiene & Tropical Medicine), Professor Peter Brocklehurst (University of Oxford), Dr Robert Tasker (University of Cambridge), and Dr Martin Ward Platt (University of Newcastle).

**Who has reviewed the study?**

The study has been approved by the North West Research Ethics Committee. It also has the support of the Chief Investigators and Chair of the Trial Steering Committee for each trial, and from the Clinical Director for each neonatal unit.

**What if there is a problem?**

If you decide to take part in the BRACELET Study and you have a concern about any aspect of the research, you could talk to Claire Snowdon or Diana Elbourne (contact details below).

If you feel that you have been harmed in some way during the research study there are no special compensation arrangements. If you are harmed and this is due to someone’s negligence then you may have grounds for legal action for compensation against the London School of Hygiene and Tropical Medicine, but you may have to pay your legal costs.

**Any queries?**

If you have any queries, please contact Claire Snowdon or Diana Elbourne who will be happy to answer any questions:

Claire Snowdon Diana Elbourne

Centre for Family Research Medical Statistics Unit

Free School Lane London School of Hygiene & Tropical Medicine

University of Cambridge Keppel Street

Cambridge CB2 3RF London WC1E 7HT

Tel: 01223 334508 Tel: 020 7927 2629

Email: [cms1000@cam.ac.uk](mailto:cms1000@cam.ac.uk) Email: [diana.elbourne@lshtm.ac.uk](mailto:diana.elbourne@lshtm.ac.uk)

**Thank you for taking the time to read this information**
